# Highly expression of Tim-3 on HIV-specific T cells associated with disease progression and T-cell exhaustion in HIV-1 infected Chinese

**DOI:** 10.1186/1742-4690-9-S2-P270

**Published:** 2012-09-13

**Authors:** Z Liu, K Hong, M Jia, J Hao, Z Gao, S Liu, Y Ruan, H Xing, Y Shao

**Affiliations:** 1 National Center for AIDS Control and Prevention, China CDC, Beijing, China

## Background

The exact mechanism of T-cell exhaustion remains to be defined during HIV-1 infection. Recent studies suggest that the inhibitory receptor T-cell immunoglobulin domain and mucin domain 3(Tim-3) may play an important role in the exhaustion of HIV-specific T cells.

## Methods

72 HIV-1 infected individuals with different disease outcomes were recruited in this study. Tim-3 expression and the profile of cellular immune response were measured by using Multicolor Intracellular Cytokine Staining (ICS) assay. Association between Tim-3 expression levels and disease progression was analyzed. And the potential role of Tim-3 on immune regulation during HIV-1 infection was investigated through assessment of CTL response with frequencies of Tim-3 expression and blocking effect.

## Results

Tim-3 was found to be highly expressed on HIV-specific CD4+ T cells and CD8+ T cells, especially in primary infectors and AIDS patients. The frequencies of Tim-3 expression correlate with disease progression. The level of Tim-3 expression was related with cytokines production and blockade of Gal-9/TIM-3 signaling partially restored the ability of HIV-specific T cells to secrete cytokines in vitro.

## Conclusion

The frequencies of Tim-3 expression correlate with disease progression in HIV-1 infected individuals and manipulating Gal-9/TIM-3 signaling pathway may provide a novel therapeutic measure to control HIV-1 replication.

